# Gene-environment-gut interactions in Huntington's disease mice are associated with environmental modulation of the gut microbiome

**DOI:** 10.1016/j.isci.2021.103687

**Published:** 2021-12-24

**Authors:** Carolina Gubert, Chloe Jane Love, Saritha Kodikara, Jamie Jie Mei Liew, Thibault Renoir, Kim-Anh Lê Cao, Anthony John Hannan

**Affiliations:** 1 Florey Institute of Neuroscience and Mental Health, Melbourne Brain Centre, University of Melbourne, Parkville, VIC 3010, Australia; 2 Melbourne Integrative Genomics, School of Mathematics and Statistics, University of Melbourne, Parkville, VIC 3010, Australia; 3 Department of Anatomy and Neuroscience, University of Melbourne, Parkville, VIC 3010, Australia

**Keywords:** Disease, Microbiology, Microbiome

## Abstract

Gut dysbiosis in Huntington's disease (HD) has recently been reported using microbiome profiling in R6/1 HD mice and replicated in clinical HD. In HD mice, environmental enrichment (EE) and exercise (EX) were shown to have therapeutic impacts on the brain and associated symptoms. We hypothesize that these housing interventions modulate the gut microbiome, configuring one of the mechanisms that mediate their therapeutic effects observed in HD. We exposed R6/1 mice to a protocol of either EE or EX, relative to standard-housed control conditions, before the onset of gut dysbiosis and motor deficits. We characterized gut structure and function, as well as gut microbiome profiling using 16S rRNA sequencing. Multivariate analysis identified specific orders, namely Bacteroidales, Lachnospirales and Oscillospirales, as the main bacterial signatures that discriminate between housing conditions. Our findings suggest a promising role for the gut microbiome in mediating the effects of EE and EX exposures, and possibly other environmental interventions, in HD mice.

## Highlights

• Gastrointestinal structure and motility are intact at an early stage in a HD mouse model
• There is sexual dimorphism in the presentation of the HD gut dysbiosis phenotype
• Bacteroidales, Lachnospirales and Oscillospirales bacteria are affected by experience
• Environmental enrichment and exercise may modulate HD via the microbiota-gut-brain axis

## Graphical abstract

**Figure:**
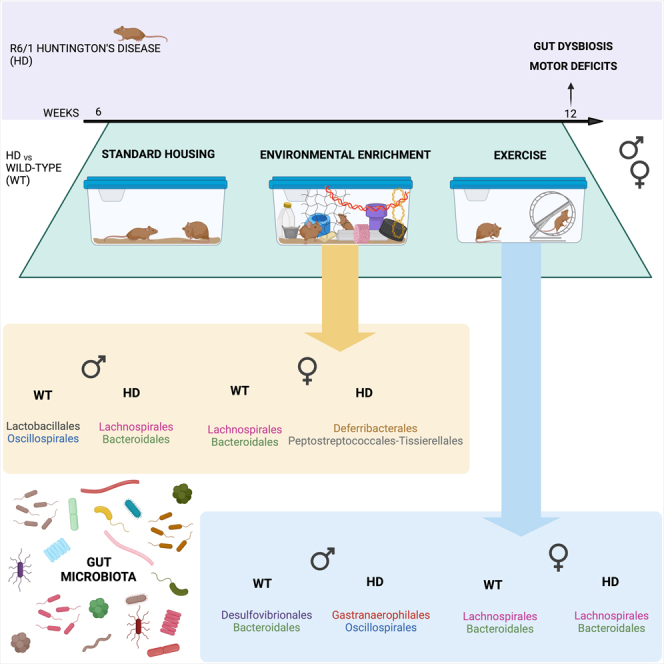

## Introduction

Huntington's disease (HD) is a neurodegenerative disorder involving complex symptomatology, including progressive cognitive, psychiatric and motor deficits (McColgan and Tabrizi, 2018). There are no disease-modifying therapies available for this devastating disease, which usually progresses over 10 to 20 years before death (Wyant et al., 2017). It is caused by the expansion of the trinucleotide (CAG) tandem repeat in the *huntingtin* (HTT) gene that encodes an expanded polyglutamine tract in the huntingtin protein (Schulte and Littleton, 2011). The R6/1 mouse model of HD expresses a human huntingtin exon 1 transgene with the CAG-repeat expansion, driven by the human huntingtin promoter, and has been shown to provide an accurate adult-onset disease model. This fragment expressed includes the polyglutamine stretch, exhibiting construct and strong face validity, including the development of behavioral, cellular and molecular deficits closely modeling HD (Nithianantharajah et al., 2008; Spires et al., 2004; van Dellen et al., 2000).

Huntingtin is expressed ubiquitously throughout the body, affecting both the brain and periphery (Li et al., 1993), including along the gastrointestinal (GI) tract (Moffitt et al., 2009; Sathasivam et al., 1999). Non-neurological symptoms associated with GI dysfunction represent significant complications in HD patients, including weight loss, constipation and nutrient deficiency, that ultimately affect a patient's quality of life (Mochel et al., 2007; Nance and Sanders, 1996; van der Burg et al., 2017). GI abnormalities were previously demonstrated in the juvenile-onset R6/2 HD mouse model, with impairment of gut motility and malabsorption of food inversely correlated with body weight (van der Burg et al., 2011). Gut permeability and decreased colon length were also seen in R6/2 HD mice (Stan et al., 2020), supporting the role of GI dysfunction in HD pathology and highlighting the need for further GI studies in adult-onset HD models.

Furthermore, evidence of GI microbial population imbalance (gut dysbiosis) was recently found in R6/1 HD mice by profiling the gut microbiome (Kong et al., 2018), showing an increase in Bacteroidetes and a proportional decrease in Firmicutes in the HD gut microbiome. These findings were subsequently replicated in R6/2 HD mice (Stan et al., 2020). Microbial dysbiosis occurs when the diverse and homeostatic microbiota profile of the gut moves into an out-of-balance or pathogenic profile, resulting in overall harmful effects on the body, with implications for neurodegenerative diseases (Patterson et al., 2014; Quigley, 2017; Tremlett et al., 2017). These preclinical findings were followed by the gut microbiome characterization in gene-positive HD subjects (Du et al., 2021; Wasser et al., 2020), not only showing the first clinical evidence of gut dysbiosis in HD but also demonstrating associations with cognitive performance and clinical outcomes (Wasser et al., 2020). Together, these studies uncover a potential new avenue to explore GI dysfunction and gut dysbiosis as targets for novel therapeutic interventions.

Environmental enrichment (EE), which enhances cognitive stimulation and physical activity, as well as physical exercise (EX) by itself, are key environmental interventions that have been established as therapeutic in R6/1 HD mice by delaying the onset and progression of disease (Pang et al., 2006; van Dellen et al., 2000). Furthermore, epidemiological evidence has implicated environmental factors as modifiers of the clinical onset of HD (Trembath et al., 2010); however, the underlying mechanisms are not well understood. In the context of neurodegenerative disorders, there is established research on the effects of various lifestyle factors, such as exercise, on GI dysfunction and the gut microbiome (reviewed in (Gubert et al., 2020; Gubert and Hannan, 2021)), yet only two studies to date have explored the effects of EE on the gut microbiome (Bice et al., 2017; Singh et al., 2019). Understanding the gene-environment-gut interactions in HD mice could inform novel therapeutic approaches, with implications for beneficial effects in other dysbiosis-related neurodegenerative diseases.

Therefore, we hypothesized that EE and EX modulate gut microbiome dysbiosis in mice, configuring one of the mechanisms that mediates the beneficial effects of extended EE and EX exposure in HD. We exposed R6/1 HD mice to a six-week protocol of either EE or EX, relative to standard-housed (SH) control conditions, before the onset of gut dysbiosis and motor deficits. Furthermore, we characterized general gut health by evaluating gastrointestinal parameters and gut microbiome profiling using 16S rRNA sequencing, to investigate the effects of these environmental interventions on both HD and wild-type (WT) mice, of both sexes.

## Results

### HD mice had decreased body weight and exercise differentially affected body weight over time in HD and WT mice

**Figure 1:**
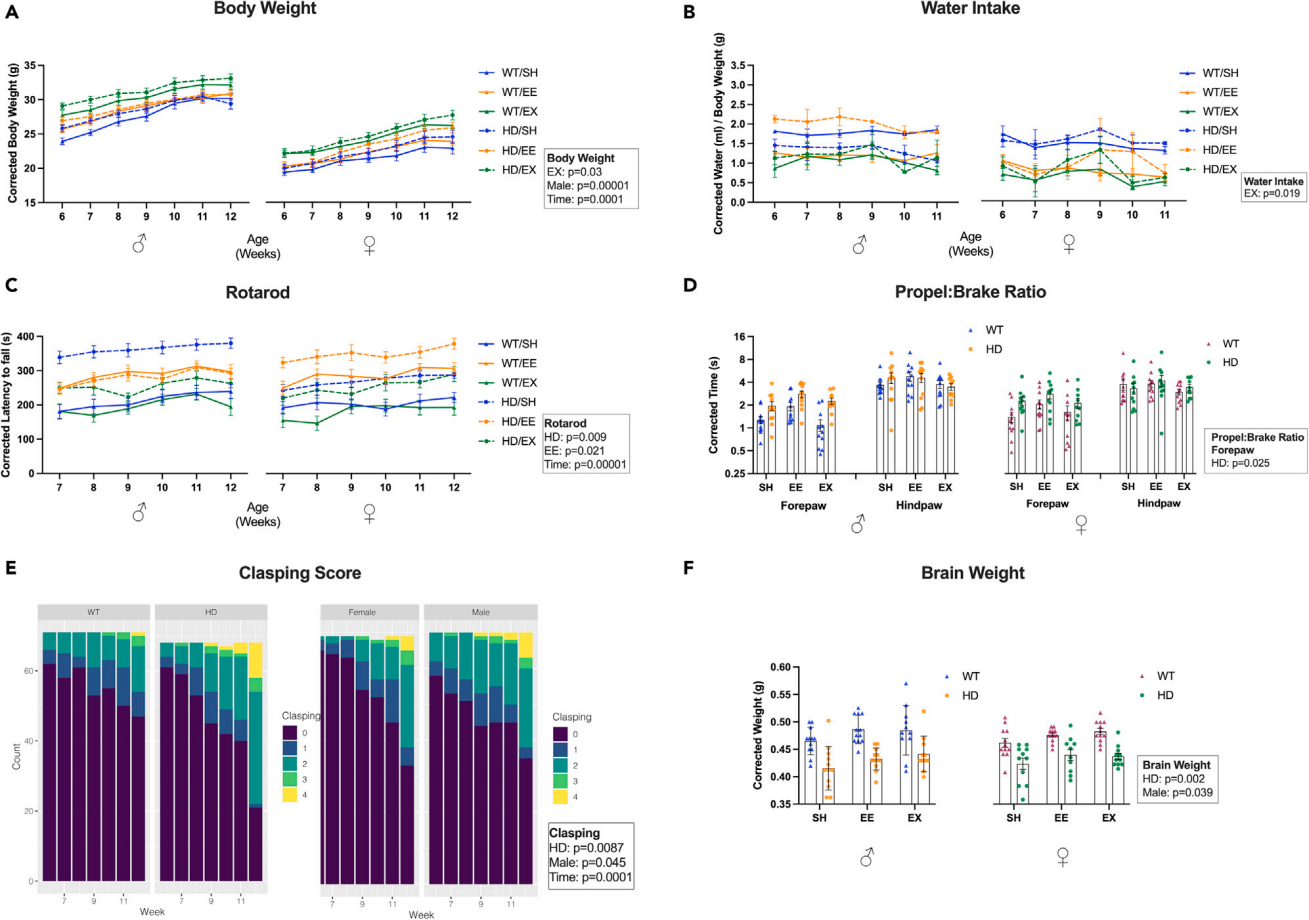
Effects of housing, genotype and sex for body weight, water intake, motor measurements, and brain weight (A) EX mice weighed more than SH mice, males weighed more than females, LMM, n = 11-12 mice. (B) EX mice drank less water than SH mice, LMM, n = 3 cages per group. (C) On average, HD mice performed better on rotarod than WT, EE mice performed better than SH and EX, LMM, n = 11 to 12 mice. (D) HD mice showed an increase in forepaw propel-to-brake ratio at 12 weeks of age than WT mice, LMM, n = 10 to 12 mice. (E) HD mice showed a greater clasping score than WT, and males had a greater clasping score than females and the score increased as the mice aged, cumulative linear mixed models, n = 11 to 12 mice. (F) At 12 weeks of age, males showed a heavier brain than females, HD mice showed a decrease in brain weight in comparison to WT, LMM, n = 10 to 12 mice. Data were corrected for interaction effects. Mean ± standard error of the mean (SEM) are represented, WT, wild-type; HD, Huntington's disease; SH, standard housing; EE, environmental enrichment; EX, exercise; LMM, linear mixed model.

Across time, EX mice weighed more than SH mice (beta = 2.709, p = 0.030) and male mice weighed more than females (beta = 6.267, p = 3.398e-06). In addition, post-hoc tests showed that most of the contrasts between different sexes were significant across all weeks, with weights in males higher than in females (Table S1). When investigating the effect of time overall, mice increased in weight as they aged (beta = 0.820, p = 2.483e-35). However, a genotype∗time interaction in linear mixed models (LMM) showed that HD mice lost weight as they aged (beta = −0.218, p = 0.0002), and housing∗time interactions showed that both EE (beta = −0.256, p = 0.0003) and EX (beta = −0.443, p = 4.452e-10) groups also lost weight as they aged (Figure 1A).

### Mice subjected to EE and EX, as well as HD mice, drank more water as they aged

A significant housing∗time interaction in LMM showed that EE mice (beta = 0.074, p = 0.029) and EX mice (beta = 0.125, p = 0.0003) drank more as they aged, but overall EX (beta = −0.797, p <0.019) housed mice drank less water in comparison to the SH mice. As they aged, a significant genotype∗time interaction effect demonstrated that HD mice had an increased water consumption in comparison to WT (beta = 0.066, p = 0.017) and a significant sex∗time interaction effect demonstrated that males drank less than females as they aged (beta = −0.084, p = 0.003). In addition, we found a significant genotype∗sex effect with HD male mice showing a decrease in water intake (beta = −0.313, p = 0.029) (Figure 1B). Post-hoc pairwise comparisons showed that for females, HD mice in SH drank more water than WT mice in EE housing across all weeks (all p-values less than 0.049). In addition, from week 7 onward, male HD mice in SH drank less water on average than female HD mice in SH (all p-values less than 0.049, Table S2).

### HD mice performed worse on the rotarod as they aged

HD mice performed better on the rotarod than WT (beta = 67.587, p = 0.021). We observed an overall improvement in rotarod performance as the mice aged (beta = 8.905, p = 0.0009); however, when assessing the interaction effect between genotype and time, we found that HD mice performed worse than WT mice as they aged (beta = −14.385, p = 3.109e-09) (Figure 1C). Overall, EE mice performed better on the rotarod than the SH group (beta = 78.859, p = 0.021). All pairwise comparisons within same sex showed better rotarod performance in WT mice in EE housing compared to WT mice in EX housing with the exception in females for week 12 only (all p-values less than 0.042, Table S3).

### EE and EX had no effect on gait deficits in HD mice

HD mice had an increased forepaw propel-to-brake ratio compared to WT mice (forepaw beta = 0.895, p = 0.025) (Figure 1D). For both sexes, the post-hoc test showed that this increase was significant only in SH and EE housing (Table S4). An increase in both forepaw and hindpaw swing time (forepaw beta = 0.015, p = 0.042, hindpaw beta = 0.011, p = 0.049) in comparison to WT counterparts was observed in LMM (Figure S1A). Post-hoc tests confirmed that the forepaw swing time was higher for HD mice compared to WT in females housed in SH (p = 0.041) (Table S5), but both sexes in SH had increased hindpaw swing time in HD mice compared to WT (Table S6). We observed no effects of housing, genotype, or sex on forepaw or hindpaw stride time (Figure S1B). In assessing gait symmetry, HD mice showed a decrease in forepaw stance width in comparison to WT (beta = −0.258, p = 0.009) and we observed a sex effect with male mice showing a greater forepaw stance width than females (beta = 0.219, p = 0.022) (Figure S1C). Among males housed in SH, HD mice had lower forepaw stance width than the WT (p = 0.036) (Table S7). HD mice showed an increase in forepaw stride length in comparison to WT (beta = 0.432, p = 0.043). The increased forepaw stride length in HD mice compared to WT mice was seen among females housed in SH (p = 0.043) (Table S8). EX mice also showed an increased hindpaw stride length in comparison to SH (beta = 0.445, p = 0.036), whereas a significant genotype∗housing interaction showed that HD exercising mice had a decrease in hindpaw stride length overall (beta = −0.538, p = 0.031) angle (Figure S1D). The increased hindpaw stride length in EX mice compared to SH was only seen in WT mice (p = 0.049) (Table S9). No differences were seen in forepaw or hindpaw absolute paw angle (Figure S1E).

### HD mice showed increased clasping score and EE and EX did not prevent this outcome

We observed a time effect with an overall increase in clasping score as the mice aged (beta = 0.413, p = 5.579e-22). As they aged, HD mice showed an increase in clasping score in comparison to WT (beta = 0.805, p = 0.009) and males showed an increase in clasping score in comparison to females (beta = 0.612, p = 0.045) (Figure 1E). No difference was found among the housing conditions (Figure S1F). Regardless of the housing condition or week, same sex mice showed a higher clasping score in HD mice than the WT mice (Table S10).

### HD and female mice have a decreased brain weight

At 12 weeks of age, HD mice showed a significantly reduced brain weight in comparison to WT (beta = −0.049, p = 0.002), and a significant sex effect demonstrated that males had a greater brain weight than female counterparts (beta = 0.030, p = 0.039). We found no effect of housing interventions on brain weight (Figure 1F). Post-hoc comparisons in females housed in SH and EE showed lower brain weight in HD mice compared to WT. In addition, in SH condition, HD mice showed higher brain weight in males than females (Table S11).

### Neither housing, genotype nor sex affected food intake

We found no differences in food intake across time (Figure S2A).

### Fecal output increases as the mice age, however no effect on housing, genotype nor sex were observed in fecal water content

**Figure 2:**
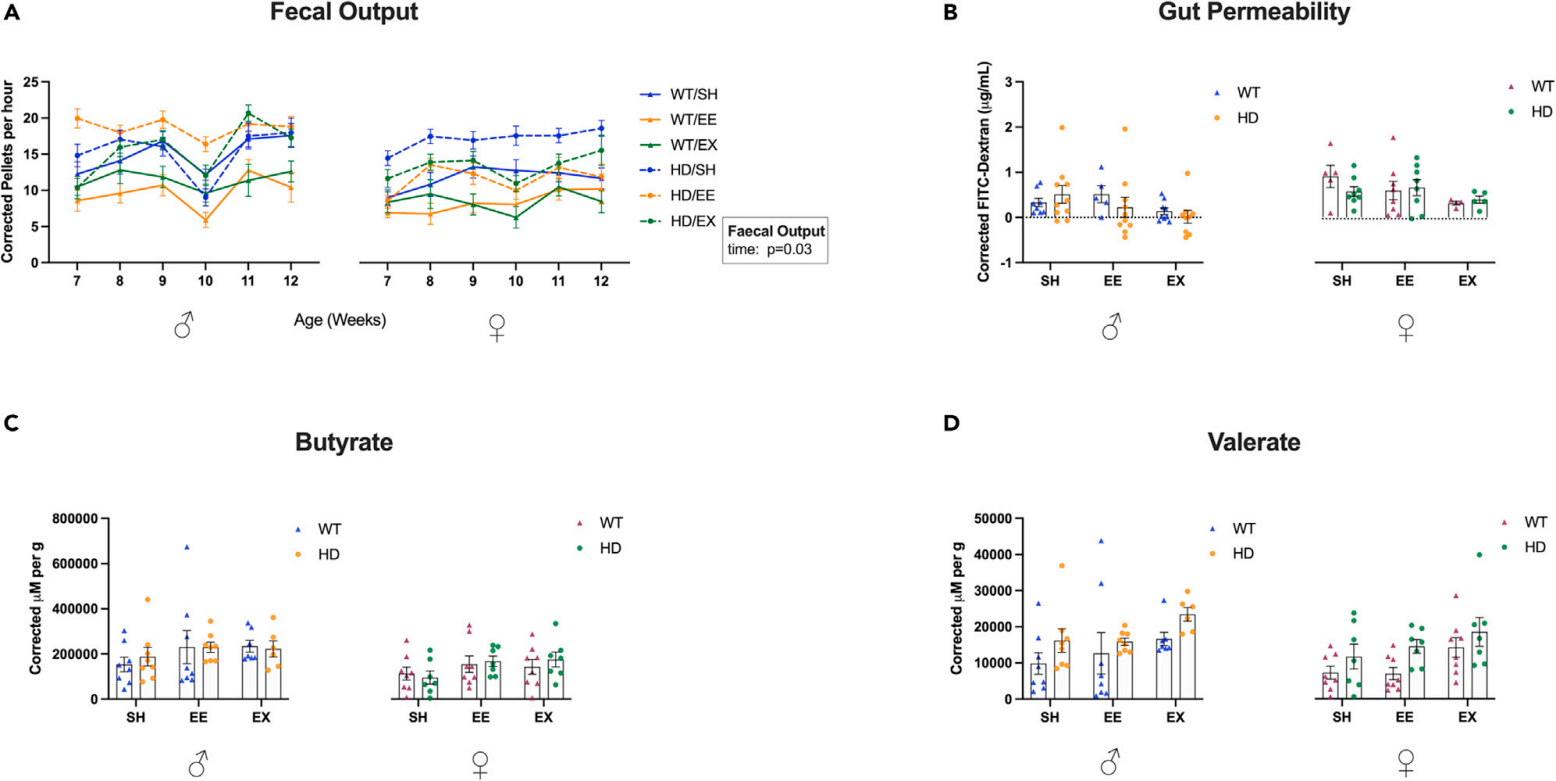
Effects of housing, genotype and sex on fecal output, gut permeability and SCFA concentrations (A) Fecal output increased with age, LMM, n = 11 to 12 mice. (B) No effect of housing, genotype nor sex on gut permeability was observed at 12 weeks of age, LMM, n = 4 to 10 mice. (C) No main effects (i.e., sex, genotype, housing) influence SCFA butyrate concentrations, LMM, n = 7 to 8 mice. (D) No main effects (i.e., sex, genotype, housing) influence SCFA valerate concentrations, LMM, n = 7 to 8 mice. Data were corrected for interaction effects. Mean ± SEM are represented. WT, wild-type; HD, Huntington's disease; SH, standard housing; EE, environmental enrichment; EX, exercise; LMM, linear mixed model; SCFA, short-chain fatty acids.

A main effect of time showed that as all the groups aged, there was an increase in fecal output (beta = 0.457, p = 0.033) (Figure 2A). There were no significant differences between groups when analyzing the fecal water content across time (Figure S2B).

### No housing, genotype nor sex effects were identified on gut transit time, gut macroscopy nor gut permeability

At 12 weeks of age, no effects of housing, genotype nor sex were seen in gut transit time, or gut macroscopy measures of colon length, caecum length and caecum weight (Figure S2C–S2F). Furthermore, we found no difference in measures of gut permeability (Figure 2B).

### Male EX mice showed decreased fecal SCFA butyrate and valerate concentrations

Short-chain fatty acids (SCFA) and branched-chain fatty acids (BCFA) are both microbiota-derived metabolites, produced by the fermentation of dietary fiber and amino acids, respectively. We investigated their fecal concentration in this study to verify the potential mediator effect that these metabolites could be playing in the effect of the environmental interventions in HD. A significant housing∗sex effect showed male EX mice have a decrease in butyrate (beta = −161105.800, p = 0.043) and valerate concentrations (beta = −12036.940, p = 0.027) (Figures 2C and 2D). Post-hoc tests showed that EX male mice had decreased butyrate and valerate concentrations (Tables S12 and S13). No effects of housing, genotype nor sex were observed in fecal SCFA concentrations of acetate, propionate or caproate, nor were there any differences observed in fecal BCFA concentrations of isobutyrate, 2-methylbutyrate or isovalerate concentrations (Figures S3A–S3F).

### The gut microbiome composition differs between genotype, sex and housing conditions

**Figure 3:**
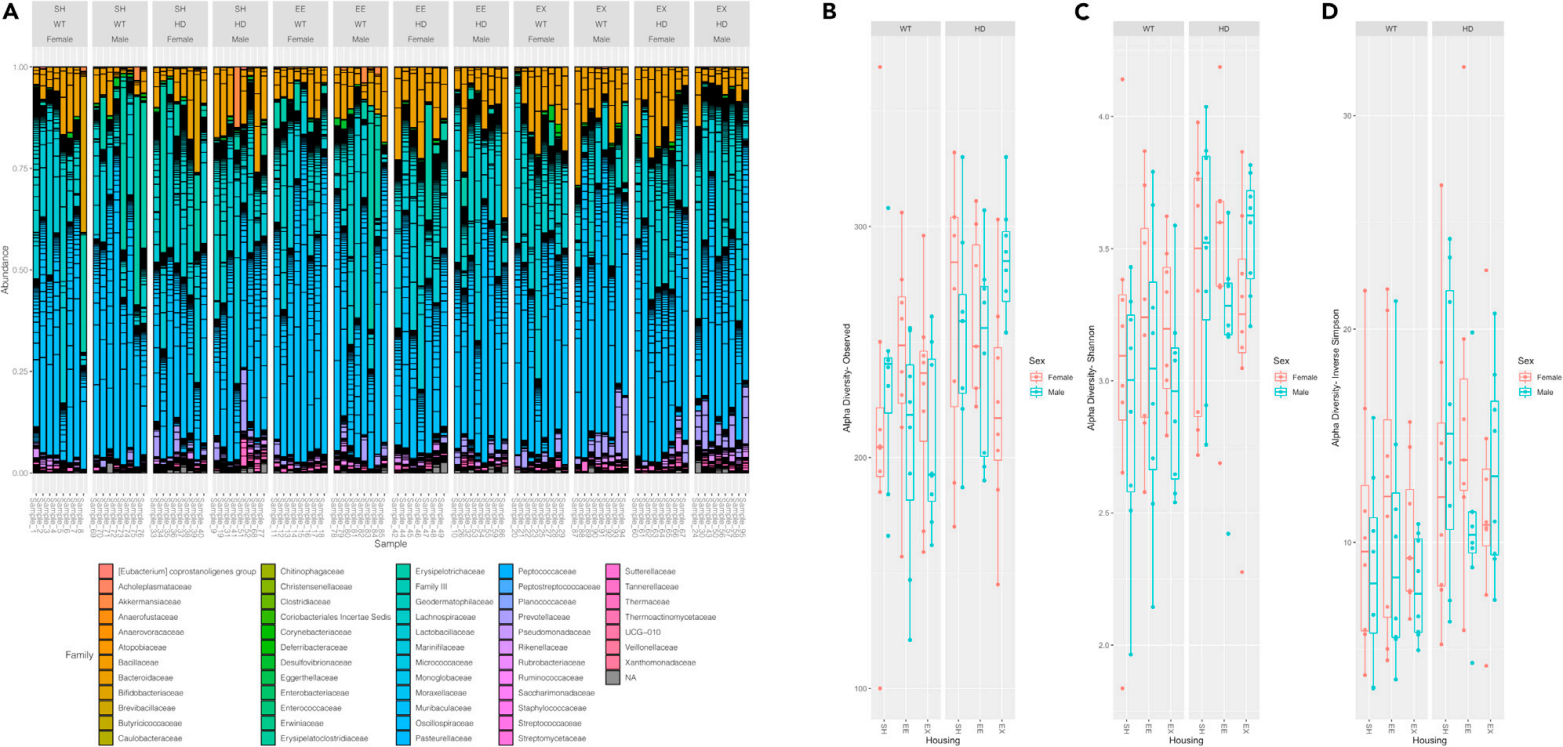
Composition bar plots for relative abundance data and microbiota alpha-diversity profiling in HD mice (A) Microbial composition at the Family level for each sample by its relative abundance. Alpha-diversity metrics including (B) Observed, (C) Shannon, and (D) Inverse Simpson (InvSimpson) indices probed for the two genotypes among different housing conditions and sex. ANOVA test indicated an increase in all three indices between HD and WT mice. n = 7 to 8 samples per group. Boxplots show median, interquartile range, lowest value, highest value and outliers. WT, wild-type; HD, Huntington's disease; SH, standard housing; EE, environmental enrichment; EX, exercise; ANOVA, analysis of variance.

To characterize the effect of housing conditions on the gut microbiome in HD mice, the genomic DNA was extracted for 16S amplicon sequencing from feces collected at 12 weeks of age, after 6 weeks of exposure to the assigned housing conditions. We detected 55 different families in the mouse microbiome samples (Figure 3A). Description of operational taxonomic units (OTUs) at the family level and higher taxonomic levels can be found in the supplementary files (Table S16). Of note, OTUs are basic units used in numerical taxonomy, being used to classify groups of closely related individuals and can refer to any taxonomic level (e.g., species, genus, family and class). Further bioinformatics analysis was performed to estimate alpha and beta diversity, which indicates differences within and between microbial communities, respectively. Specifically, alpha diversity counts the total number of species in an ecosystem (within the community), summarizing the structure of the community regarding the number of taxonomic groups. Beta diversity evaluates the extension of change (between the community), defining the degree of the microbiome differentiation. We probed the indices for the two genotypes (WT vs. HD) among the different housing conditions (SH, EE, and EX) and sex (males, females).

The alpha diversity metrics used include Observed (Figure 3B), the species richness measure representing the number of observed OTUs in a sample, Shannon (Figure 3C) and Inverse Simpson (InvSimpson) (Figure 3D) indices, which consider both richness and the relative abundance of OTUs (i.e., evenness). Further statistical testing using analysis of variance (ANOVA) revealed no significant differences in any of these indices between sex or housing conditions (Figures 3B–3D). We found that HD mice have higher indices of alpha diversity in comparison with WT mice [ANOVA, Observed (F = 11.45, df = 1, p = 0.00105) Figure 3B; Shannon (F = 13.03, df = 1, p = 0.000497) Figure 3C; Inverse Simpson (F = 9.341, df = 1, p = 0.00292) Figure 3D].

**Figure 4:**
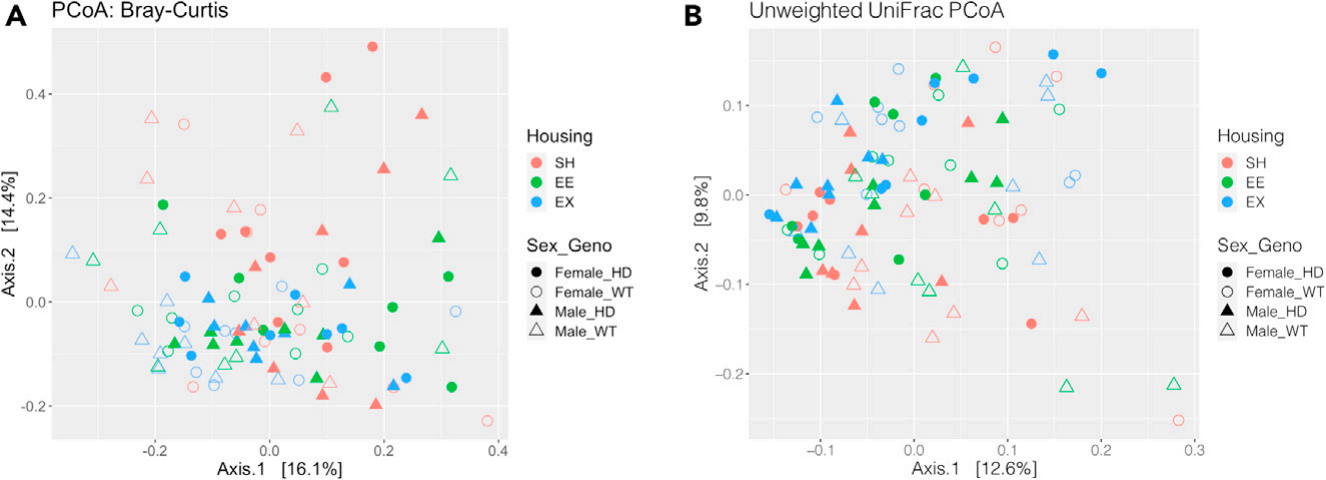
PCoA plots of beta-diversity profiling based on housing, genotype and sex There were significant differences between genotypes, housing and sex in both (A) Bray-Curtis and unweighted (B) UniFrac distances (permutation multivariate ANOVA with 999 permutations). n = 7 to 8 samples per group. WT, wild-type; HD, Huntington's disease; SH, standard housing; EE, environmental enrichment; EX, exercise; ANOVA, analysis of variance; PCoA, principal coordinate analysis.

For beta diversity measurements we used both Bray-Curtis (accounts for the abundance of the OTUs) and unweighted UniFrac (accounts for the phylogenetic relationship between the OTUs) distance. The entire cohort was examined together in a principal coordinate analysis (PCoA) plot (Figures 4A and 4B). Although these plots did not visually highlight strong clustering according to any factor of interest, these distances were found to be statistically different for genotype, housing, and sex (Figure 4A, Bray Curtis dissimilarity distance Permanova (genotype) R^2^ = 0.03, p = 0.001; Permanova (housing) R^2^ = 0.04, p = 0.002; Permanova (sex) R^2^ = 0.02, p = 0.02; Figure 4B, unweighted UniFrac distance Permanova (genotype) R^2^ = 0.02, p = 0.004; Permanova (housing) R^2^ = 0.03, p = 0.002; Permanova (sex) R^2^ = 0.02, p = 0.005).

**Figure 5:**
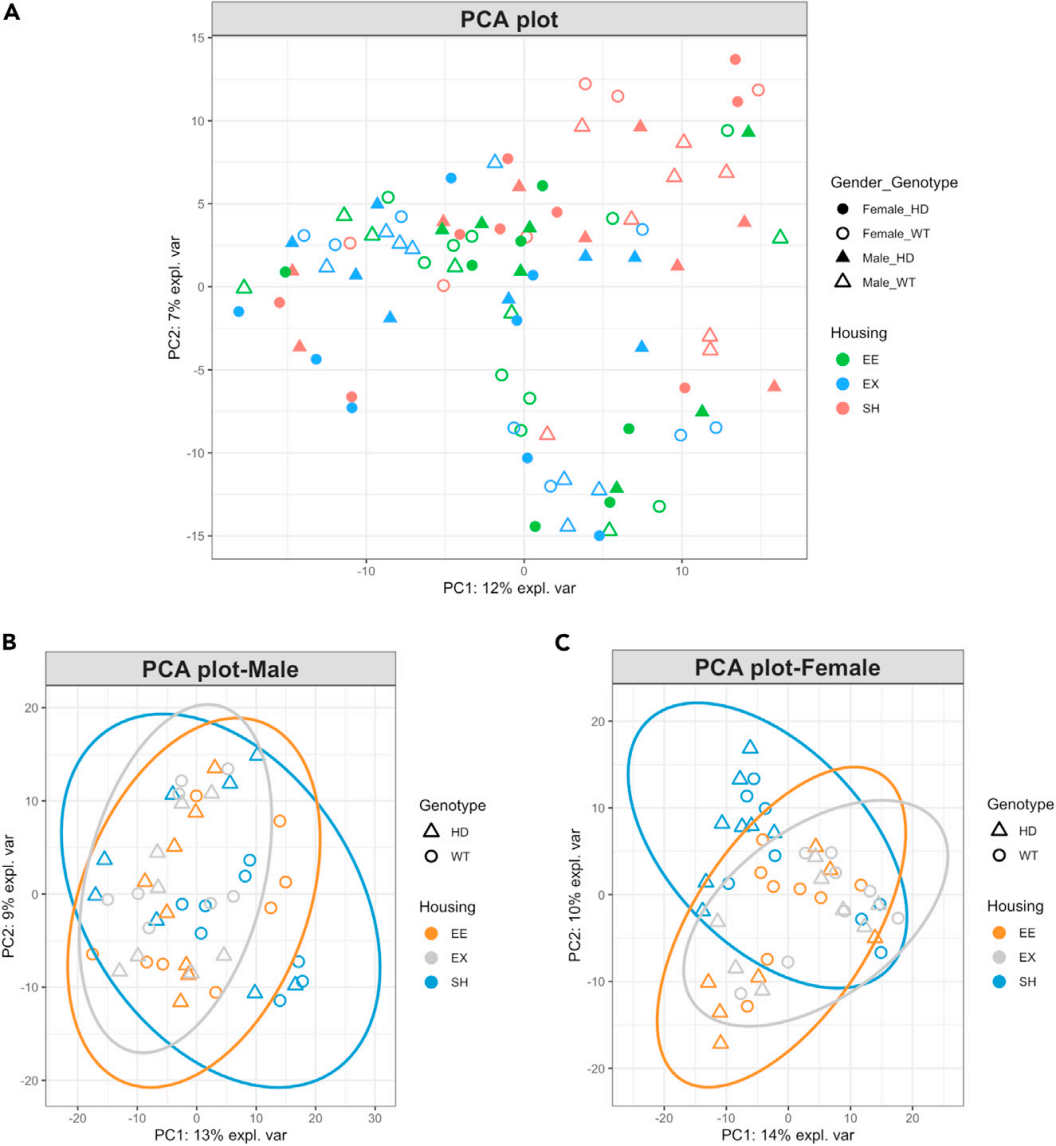
PCA sample plots of housing, genotype and sex (A) PCA of the entire cohort did not show any clustering of samples according to sex. PCA separated for (B) males and (C) females with colors and symbols indicating housing and genotype: samples tend to cluster according to housing conditions. n = 7 to 8 samples per group. PCA, principal component analysis; WT, wild-type; HD, Huntington's disease; SH, standard housing; EE, environmental enrichment; EX, exercise.

Because we observed sex differences in the beta diversity distances, we separated the sexes for the subsequent analysis. Furthermore, the entire cohort was examined in a Principal Component Analysis (PCA) (Figure 5A), and sexes were then separated and visualized with PCA (Figures 5B and 5C).

**Figure 6:**
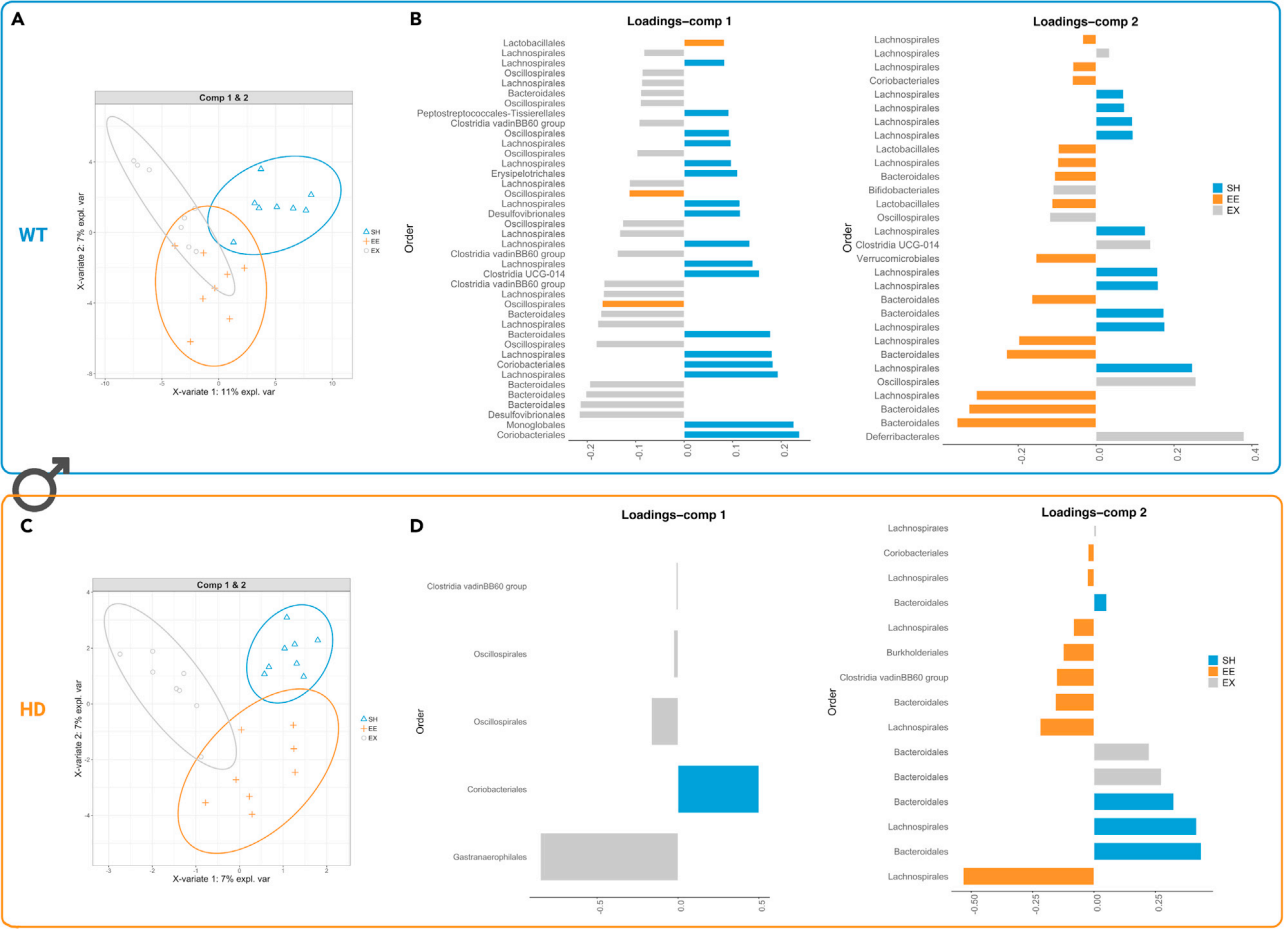
Identification of a bacterial signature in males discriminating housing conditions with sPLS-DA Sample plots with 0.95 confidence ellipse plots show discrimination between housing conditions for (A) WT males and (C) HD males. Contribution plots for (B) WT and (D) HD show the top OTU features indicated at the Order level selected on the first and second components. The most discriminative OTUs are ordered from bottom to top, where the length of the bars represents the regression coefficient assigned to each selected OTU from sPLS-DA, and colors represent the housing condition that has maximum median value. Classification error rates resulting from five-fold cross-validation repeated 10 times were 0.47 for males (all housing and genotypes), 0.45 for male WT (housing condition), 0.34 for male HD (housing condition). Specifically, per housing condition SH, EE, and EX, the error rates were 0.28, 0.68, 0.45 for male WT and 0.39, 0.39, 0.25 for male HD. n = 7 to 8 samples per group. WT, wild-type; HD, Huntington's disease; SH, standard housing; EE, environmental enrichment; EX, exercise; OTU, Operational taxonomic units; sPLS-DA, sparse partial least squares regression-discriminant analysis.

**Figure 7:**
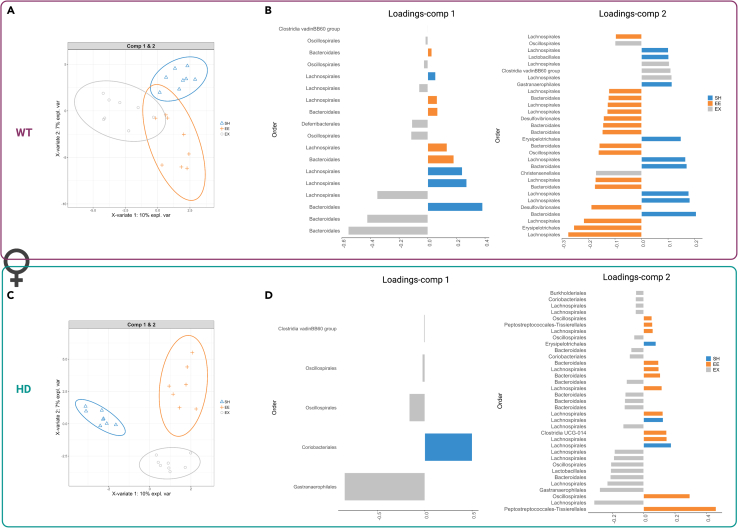
Identification of a bacterial signature in females discriminating housing conditions with sPLS-DA Sample plots with 0.95 confidence ellipse plots show discrimination between housing conditions for (A) WT females and (C) HD females. Contribution plots for (B) WT and (D) HD show the top OTU features indicated at the Order level selected on the first and second components. The most discriminative OTUs are ordered from bottom to top, where the length of the bars represents the regression coefficient assigned to each selected OTU from sPLS-DA, and colors match the housing condition that has maximum median value. Classification error rates resulting from five-fold cross-validation repeated 10 times were 0.50 for females (all housing and genotypes), 0.38 for female WT (housing condition), 0.38 for female HD (housing condition). Specifically, per housing condition SH, EE, and EX, the error rates were 0.29, 0.51, 0.34 for female WT and 0.20, 0.53, 0.44 for female HD. n = 7 to 8 samples per group. WT, wild-type; HD, Huntington's disease; SH, standard housing; EE, environmental enrichment; EX, exercise; OTU, Operational taxonomic units; sPLS-DA, sparse partial least squares regression-discriminant analysis.

Next, to further investigate the specific OTUs which could contribute to the stratification of samples according to their housing conditions, we used Sparse Partial Least Squares regression - Discriminant Analysis (sPLS-DA) (Lê Cao et al., 2011), which is a multivariate method to identify microbial drivers discriminating phenotype groups. In this study, we identified microbial drivers discriminating housing conditions among different sex and genotype (Figures S4A and S4B). The loading values can be found in Tables S14 and S15. We identified a signature of bacteria discriminating housing conditions (SH, EE, EX) in male WT (Figure 6A) and HD mice (Figure 6C), as well as in female WT (Figure 7A) and HD mice (Figure 7C), as shown at the OTU level in their respective plots (Figures 6B, 6D, 7B, and 7D). The Bacteroidales, Lachnospirales and Oscillospirales orders were the main bacterial signatures that discriminate the housing conditions. For the male WT mice, we identified Coriobacteriales and Monoglobales (in SH housing), Oscillospirales and Lactobacillales (in EE housing), and Desulfovibrionales and Bacteroidales (in EX housing). In the male HD mice, we identified Coriobacteriales, and Bacteroidales (in SH housing), Lachnospirales and Bacteroidales (in EE housing), and Gastranaerophilales and Oscillospirales (in EX housing). For female WT mice (for SH, EE, and EX) we identified Bacteroidales and Lachnospirales and for the HD mice Bacteroidales and Lachnospirales (in SH housing), Deferribacterales and Peptostreptococcales-Tissierellales (in EE housing), and Lachnospirales and Bacteroidales (in EX housing).

LMM analysis did not highlight any significant OTUs after correction for multiple testing at the 0.05 significance level (Tables S14 and S15) However, at a significance level of 0.1, one OTU that belonged to *Oscillibacter* Genus and *Oscillospiraceae* family showed a significant difference among genotypes in males.

## Discussion

This is the first study to fully evaluate gastrointestinal function in an adult-onset HD animal model and to investigate gene-environment interactions associated with gut dysbiosis in HD mice. We reproduced previous findings showing that HD mice have a differential alpha–diversity (Kong et al., 2018) and also found effects of genotype, housing and sex in beta diversity metrics, indicating modulatory effects on the gut microbiome composition in these mice. sPLS-DA analysis identified the orders Bacteroidales, Lachnospirales, and Oscillospirales as the main bacterial signatures that discriminate between housing conditions with further specificities between housing, genotype and sex, suggesting a promising role of the gut microbiome as a mediator for the positive effects of extended EE and EX exposure in HD mice.

We found a higher alpha diversity for all metrics analyzed in HD mice compared to WT mice, similarly to Kong et al., 2018. An increased number of species and higher evenness is commonly associated with a healthier bacterial environment, with the community being more resilient to new species, among other characteristics (Lozupone et al., 2012). Neurodegenerative disorders including Alzheimer's, Parkinson's, and motor neuron disease have been reported to be associated with lower alpha diversity in patients in comparison with controls (Keshavarzian et al., 2015; Rowin et al., 2017; Vogt et al., 2017). In Huntington's disease, a lower (Wasser et al., 2020) and higher alpha diversity (Du et al., 2021) was demonstrated in the gut microbiome of HD gene-expansion carriers (including symptomatic individuals) when compared to controls. A recent meta-analysis has indicated that alpha diversity was not associated with neurological disorders, particularly Parkinson's disease and multiple sclerosis (Plassais et al., 2021). In fact, from an ecological perspective, higher diversity is not always associated with a better outcome (Cardinale et al., 2006; Shade, 2017). Importantly, increased diversity can be associated with a reduction of stability in gut communities (Coyte et al., 2015) and recently it was demonstrated that R6/1 HD mice show heightened gut microbial volatility and perturbed gut microbiome function (Kong et al., 2021). There is also evidence that diet, body mass, gut physiology, morphology and transit time can be associated with alpha diversity (Reese and Dunn, 2018). Although we did not see any difference in food intake, HD mice lost weight as they aged (as expected) and showed an increased water intake, which are potentially related to the increase in alpha diversity.

Mouse models have a controlled environment, including diet (a major modulator of microbiota), which is not possible for clinical studies, and this might help to explain the different alpha diversity findings between clinical and preclinical HD studies. Therefore, the relationship between the gut microbiota community with the host, including the fitness of the host, should be considered while attempting to understand microbial diversity differences (Reese and Dunn, 2018).

Despite no differences in food intake, and consuming more water than WT mice, HD mice lose weight as they age. The discriminating signature of bacteria between HD and WT mice could contribute to this weight loss, by influencing host metabolism (Liot et al., 2017) and food absorption, which is inversely associated with body weight (van der Burg et al., 2017). Increased thirst in HD mice is likely because of xerostomia (dryness of the mouth), which has been established in both HD patients and mice, or because of hypothalamus neurodegeneration which is correlated with increased thirst in HD mice (Wood et al., 2008). There were no changes in fecal water content, which has been associated with microbiota composition (Vandeputte et al., 2016), despite increased water intake in HD mice. This suggests that the tightly regulated water absorption, occurring predominantly in the small intestine, is intact at this stage (Kiela and Ghishan, 2016; Masyuk et al., 2002). EE and EX groups also showed decreased body weight despite no differences in food intake and increased water intake, likely because of respective increases in physical activity.

At this early stage, we observed no differences in gut permeability, fecal water content, caecum length, caecum weight, or colon length. We observed an increase in fecal output overall with age and no differences in gut transit time. Together, these results suggest that gastrointestinal structure and motility are intact at this early stage in R6/1 HD mice, and are not influenced by housing conditions. Increased gut permeability and decreased colon length and a longer transit time have been found in juvenile-onset R6/2 HD male mice (Stan et al., 2020; van der Burg et al., 2011). At 12 weeks of age in the adult-onset R6/1 HD mouse model, it may be too early in disease progression to see significant impairments in gastrointestinal structure and motility. Furthermore, this may also indicate that gut dysfunction is not related to the development of HD symptoms but rather appears with the progression of the disease. Further studies following up slow-onset HD models until later ages should be performed to identify at what stage of the disease gut dysfunction occurs as this may enlighten the causal relationship between gut dysbiosis and gut dysfunction in HD.

When analyzing beta diversity, or the composition of the gut microbiome, we found significant changes associated with genotype, housing and sex on both indexes evaluated. This indicates that the gut microbiomes are distinguishable in abundance (Bray-Curtis index) and are phylogenetically different (unweighted Unifrac index). Previously, changes in the Bray-Curtis index distinguished genotype and unweighted UniFrac distinguished sex in R6/1 HD mice (Kong et al., 2018). The analysis of the gut microbiome of HD gene-expansion carriers (including symptomatic individuals) when compared to controls also showed differences in beta diversity, specifically unweighted UniFrac (Du et al., 2021; Wasser et al., 2020). It is important to note that, while both PCoA and PCA plots did not indicate strong visual clustering, the averaged distance-based coefficient of determination from the Bray Curtis and UniFrac results determined that microbiome variations associated with housing, genotype and sex indicated significant differences ranging from 0.02-0.04. Therefore, our findings support a subtle but significant difference in beta diversity, distinguishing microbial community composition between genotype, housing, and sex.

Unsurprisingly, we found a sex effect in the gut microbiome composition analysis, an important variable affecting the gut microbiota that is established in both animal and human studies (reviewed in (Kim et al., 2020)), demonstrating differences in microbial diversity and disease susceptibility (Ma and Li, 2019). In the context of neurodevelopmental, psychiatric, and neurodegenerative disorders, sex can play a role in shaping gut microbial communities; however, sex hormones may not be the only pathway involved (Org et al., 2016), with promising links between sex, gut microbiota, the immune system, and the brain (reviewed in (Holingue et al., 2020)). Sex differences are also observed in mouse models of irritable bowel syndrome (IBS), with males demonstrating a decreased microbiota species richness and beta diversity as measured by the unweighted UniFrac distance (Kozik et al., 2017). We also observed sex effects in measures of water intake, body weight, clasping, Digigait, and SCFA concentrations, with males drinking less, weighing and clasping more, showing an increase in brain weight and a wider stance width as well as decreased concentrations of butyrate and valerate. Sex differences in the microbiota profiles could alter metabolic by-products and lead to differential communication along microbiota-gut-brain pathways, which alongside the signature of bacteria discriminating between genotypes in both males and females, could be contributing to the sexual dimorphism in the presentation of the HD phenotype.

Previous studies have established sex differences in HD mouse models, with the R6/1 male mice showing impaired weight gain, motor coordination and spatial memory earlier than females (Reviewed in (Mo et al., 2015)). However, female HdhQ200/200 mice display earlier deterioration of locomotor and motor coordination (Cao et al., 2019). Two large cohort clinical studies have shown that women with HD present a more severe presentation of symptoms and a faster progression mainly in motor and functional domains (Zielonka et al., 2013, 2018). Together, these findings suggest a complex effect of sex on the phenotypic presentation and progression of HD in both mice and humans. Further studies exploring the complex interactions between sex and gut microbial composition are required to establish if they are linked to the sexually dimorphic characteristics seen in the HD phenotype, which could be used to personalize clinical strategies.

Considering this sex effect, we further separated our analysis based on sex using sPLS-DA to identify microbial drivers that discriminate the housing conditions. The gut microbiome signature discriminating the effect of EE in WT (males: Oscillospirales/Lactobacillales and females: Bacteroidales/Lachnospirales) and EX in WT (males: Desulfovibrionales/Bacteroidales and females: Bacteroidales/Lachnospirales) characterizes the effect of EE and EX in CBAxC57Bl/6 mice (the WT background strain used in this study), or control mice independently, with potential translation for other models. These results demonstrate the importance of studying the effect of these interventions in both sexes and how EE and EX affect gut microbiome signatures in WT mice. The effects of EE and EX on the microbiome in HD is of particular interest, to understand how these housing interventions result in beneficial effects. The gut microbiome signature discriminating the effect of EE in HD (males: Lachnospirales/Bacteroidales and females: Deferribacterales/Peptostreptococcales-Tissierellales) involves the order Lachnospirales, which is an anaerobic, fermentative producer of SCFAs and related to the production of beneficial metabolites for the host (reviewed by (Vacca et al., 2020)). The order Bacteroidales is associated with host-associated microbial communities and with members linked to the healthy gut microbiome environment (Coyne et al., 2019). Members of the order Deferribacterales are preferentially anaerobic, fermentative, and are closely associated with gut inflammation, with findings indicating that they can be a pathobiont (potentially pathogenic resident microbes) but also protective in a colitis model (Herp et al., 2019; Loy et al., 2017). The gut microbiome signature discriminating the effect of EX in HD (males: Gastranaerophilales/Oscillospirales and females: Lachnospirales/Bacteroidales) consist of the order Gastranaerophilales, which is involved in the conversion of carbohydrates into lactate, ethanol, and formate (Soo et al., 2014) and together with Lachnospirales, Oscillospirales, and Peptostreptococcales-Tissierellales are part of the class Clostridia, which are commensal bacteria involved in the maintenance of overall gut homeostasis and function (Lopetuso et al., 2013). Collectively, this analysis revealed that the gut microbiome signature differentiating the housing conditions varies between sex and genotype and uncovered promising modulators and candidate targets for enviromimetics, for both EE and EX.

Only two studies have previously explored the influence of EE on the gut microbiome. A study using a colon tumor mouse model suggested that environmental enrichment could decrease tumor growth through enhanced microbiota diversity (Bice et al., 2017), and a study using a Parkinson's disease mouse model (mice overexpressing the complete human SNCA gene) found that enrichment reduced gut inflammatory markers and was able to modify the gut microbiome composition (Singh et al., 2019). However, it is important to note that both studies included the addition of running wheels to their EE protocol, thus making it impossible to separate the effect of EE from exercise alone. Our study is the first to separate both conditions (EE and EX) and evaluate specifically the effect of environmental enrichment and exercise alone. The effect of exercise on the gut microbiome is better understood, with exercise having therapeutic effects on brain functioning (Gubert and Hannan, 2021), promoting general gut health, increasing microbial community diversity and Firmicutes phylum, SCFA production, and decreasing gut inflammation (Allen et al., 2018; Campbell et al., 2016; Hoffman-Goetz et al., 2010), with relevant implications for neurodegenerative diseases (Gubert et al., 2020).

We found a decrease in SCFA butyrate and valerate concentrations in feces of male EX mice, which was unexpected as voluntary wheel running has been previously reported to increase butyrate concentrations in rats (Matsumoto et al., 2008). This could mean that specific changes in the exercising male microbiota populations may be resulting in decreased SCFA butyrate and valerate concentrations. Butyrate plays an important role in maintaining tight junctions between epithelial cells (Peng et al., 2009), yet we found no changes in gut permeability. It is important to note that in mouse models, the relationship between EX and butyrate is not well understood, and even less so for valerate. Furthermore, we also measured SCFAs in the feces, whereas exploring SCFAs in the cecal content and plasma may provide a clearer picture as to how these by-products are involved in gut barrier structure and function.

Taken together, our data aligns with the hypothesis that HD is a whole-body disorder, with the onset of gut dysfunction starting to appear at an early stage of the disease with the potential to worsen with disease progression. Our data support previous studies indicating that gut dysbiosis can be considered a pathological feature of the disease from an early stage and therefore a potential therapeutic target. Finally, we showed that despite the relatively short exposure to EE and EX housing conditions, we uncovered a bacterial signature associated with both environmental conditions, indicating that the beneficial effects of extended exposure to these housing interventions on the onset and progression of HD symptoms could occur, at least in part, via the microbiota-gut-brain axis. The implications of our findings include the uncovering of promising gut microbiome components as candidate targets for enviromimetics, for both EE and EX. The differential effects of genotype and sex could be used to further personalize clinical strategies. Further studies are needed, with longer exposure times for both interventions, to clarify the extent to which the gut microbiome and GI system could be mediating the therapeutic effects of environmental enrichment and exercise.

### Limitations of the study

This study was performed at an early stage of the disease. We observed that the HD phenotype emerged in the R6/1 mice consistent with previous findings, including a decrease in rotarod performance as they aged, postural instability in Digigait measures, increased clasping score, and decreased brain weight (Li et al., 2005; van Dellen et al., 2000; Wright et al., 2015). Although these outcomes demonstrate that our HD model is replicating the expected phenotype, our relatively brief EE and EX interventions had not yet modulated these early signs of the disease. We did find that EE improved rotarod performance overall when compared to SH and EX groups. These improvements could be attributed to the fine motor skills developed while exploring the enriched home cage (Nithianantharajah and Hannan, 2006), which could improve grip specifically required in the rotarod task. Furthermore, in addition to motor capabilities, learning and motivation can also play a role in the rotarod performance. Although we have habituated the mice to the apparatus before the test, we did not systematically train them, and this could have potentially affected our results.

The present study was dedicated to the environmental effects on gut dysbiosis in HD associated with the early stage of the disease. It is important to note that our environmental enrichment protocol followed an exposure time of six to twelve weeks of age, with additional SuperEE from ten to twelve weeks. Following the preclinical findings of Kong et al. (2018) and 2020, demonstrating the onset of dysbiosis in HD mice at 12 weeks of age, our protocol ended at this time point to explore the effects of housing on gut dysbiosis and gastrointestinal measurements. Studies reporting housing effects on motor parameters of HD consisted of environmental exposures ranging from 10-22 weeks and superEE done over 32 weeks (Mazarakis et al., 2014; Pang et al., 2006; van Dellen et al., 2000) which may be why we did not see HD-specific motor deficits attenuated by housing conditions. In the present study, we have not found many interactions between housing and genotype, probably because of the same exposure time limitation. Interestingly, the relevant literature that showed that EE and EX could delay the onset of HD found genetic and environmental effects but did not describe interactions (Pang et al., 2006; van Dellen et al., 2000). This fact did not decrease the impact of their groundbreaking findings. Although a longer exposure to the housing conditions in HD is outside the scope of this study, this should be addressed in subsequent investigations. Furthermore, differences in running wheel activity between males and females have been previously reported in R6/1 mice (Ransome and Hannan, 2013) and another mouse model of HD (Dorner et al., 2007), therefore tracking running activity in future studies may provide a clearer insight into the effect of exercise on sex dimorphism.

## STAR★Methods

### Key resources table

**Table:** 

REAGENT or RESOURCE	SOURCE	IDENTIFIER
**Chemicals, peptides, and recombinant proteins**		

4 kDa fluorescein isothiocyanate (FITC) dextrin	Sigma-Aldrich	FD4; CAS: 60842-46-8
Carmine red dye	Sigma-Aldrich	C1022; CAS: 1390-65-4

**Critical commercial assays**		

PowerSoil HTP kit	Qiagen	https://www.qiagen.com/us/products/discovery-and-translational-research/dna-rna-purification/dna-purification/microbial-dna/dneasy-powersoil-htp-96-kit/

**Deposited data**		

The datasets and metadata related to this study have been deposited in the NCBI Sequence Read Archive	This paper	BioProject number PRJNA770470
The reproducible R code and report for the statistical analysis	This paper	https://github.com/SarithaKodikara/Gene_environment_gut_interactions_in_Huntington-s_disease

**Experimental models: Organisms/strains**		

Mouse: R6/1: B6.Cg-Tg(HDexon1)61Gpb/J	The Jackson Laboratory	JAX: 006471

**Software and algorithms**		

R (Version 4.1.0)	R Development Core Team	https://www.r-project.org/
RStudio (Version 1.4.1717)	RStudio Public-benefit corporation	https://www.rstudio.com/
Prism 9 (Version 9.3.0)	GraphPad	https://www.graphpad.com/scientific-software/prism/

**Other**		

Rotarod	Ugo Basile	https://www.ugobasile.com/products/catalogue/motory-coordination-grip-strength-activity/item/83-47650-new-mouse-rotarod-ng
Digigait	Mouse Specifics Inc	https://mousespecifics.com/digigait/
PHERAstar FSX	BMG Labtech	https://brochures.bmglabtech.com/view/96826461/6/
MiSeq platform	Illumina	https://sapac.illumina.com/systems/sequencing-platforms/miseq.html

### Resource availability

#### Lead contact

Further information and requests for resources and reagents should be directed to and will be answered by the lead contact, A.J.H (anthony.hannan@florey.edu.au).

#### Materials availability

This study did not generate new unique reagents.

### Experimental model and subject details

#### Animal husbandry, environmental enrichment, exercise and standard housing conditions

Hemizygous R6/1 male mice (on a CBAxC57Bl/6 strain background) were crossed with CBAxC57Bl/6 females to generate male and female wild-type (WT) and R6/1 (hereafter termed HD) littermates. Genotypes were characterized using genomic DNA from a tail biopsy.

The mice were randomised into either standard-housed (SH), environmental enrichment (EE) or exercise (EX) groups according to genotype and sex (3-5 mice per cage, 4 cages per group) from 6 weeks of age, to avoid any sharing of microbiota between different genotypes (mice are coprophagic and ingestion of cage-mate feces can modulate gut microbiota). All mice were exposed to the assigned housing condition for 6 weeks. The SH condition consisted of an open-top cage (34 × 16 × 16 cm) with basic sterilized wood shavings and facial tissues for bedding and nesting materials. EE and EX mice were housed in a larger cage (40 cm × 28 cm × 18 cm) with sterilized wood shavings and facial tissues for bedding and nesting materials. Additionally, EE cages contained tunnelling and climbing objects as well as objects of different materials (plastic, metal, glass, rubber and cotton ropes), textures, shapes and sizes, to enhance sensory cognitive and motor stimulation (reviewed in Nithianantharajah and Hannan, 2006). From weeks ten to twelve, EE mice were also exposed three times a week for one hour to a super-enriched condition in a larger playground arena (43 × 80 × 51 cm), containing a variety of larger enrichment objects, tunnelling and climbing apparatus of different materials (plastic, metal, glass, rubber and cotton ropes), with no wood shavings or tissues as previously described (Mazarakis et al., 2014). Each EX cage contained two running wheels (12 cm in diameter) to guarantee all mice had access to voluntary wheel running. Running wheels were excluded from the EE housing to ensure the effects of wheel-running exercise were exclusive to the EX mice. Paper, wood, and cardboard objects were not added to EE housing, so as to not influence microbiota composition via ingestion. Mouse cages and housing objects were processed through a washer at 82°C before use.

All mice had *ad libitum* access to sterilized food and filtered water (through a 0.5μm filter) and were housed in a room with a 12:12 h light/dark cycle, controlled for temperature (22°C) and humidity (45%). Cages were changed weekly to ensure novelty for the EE mice and body-weight assessment was also performed weekly. All experiments and procedures were approved by The Florey Institute of Neuroscience and Mental Health Ethics Committee and were performed following the research guidelines and regulations of the National Health and Medical Research Council.

### Method details

#### Food & water intake

Food and water intake was assessed over six weeks. Intake was normalized to body weight to account for individual weight variability. These results represent g of food per g of body weight, and ml of water per g of body weight.

#### Motor testing

##### Rotarod

The Rotarod (Ugo Basile, Varese, Italy) consists of a motorized rotating cylinder that is divided into five compartments. One mouse is placed into each compartment onto the accelerating cylinder at a constant speed of 4 rpm before the cylinder gradually accelerates to 40 rpm over 300 seconds. At 6 weeks of age mice were exposed to the apparatus under the described protocol as a habituation trial. The latency for the mice to fall onto the cushioned lever below was recorded and analysed from week 7 to 12 of age. Note that for some of the graphics, where the rotarod variable was corrected for interaction effects in the linear mixed models (see Statistical Analysis), the corrected values for latency to fall can be above 300s.

##### Clasping

Clasping is the retraction of paws during tail suspension and is a phenotypic characteristic of the HD transgenic mice, independent of motor coordination. In each session, mice were suspended from their tails for 30 seconds, while the clasping action of the paws was observed, and the highest clasping score recorded. Mice were scored on a five-point scale from 0 to 4, with a score of 0 indicating no clasping and a score of 1 to 4 indicating the number of paws clasping. The experimenter was blinded to experimental groups during scoring.

##### Digigait

At 12 weeks of age, mice were assessed using Digigait (Mouse Specifics Inc., Boston, MA, USA). The Digigait apparatus consists of a transparent testing chamber that sits above a translucent perspex treadmill belt. A digital video camera is mounted below the treadmill, to record the paws of the mice during treadmill locomotion. The mouse was placed inside the chamber and allowed to habituate for 10 to 15 seconds before the treadmill is accelerated to 15cm/s. Once the mouse was walking consistently for 2-5 seconds, 4-10 footsteps (or 3 seconds walking) are recorded and analyzed, after which the mouse was removed from the testing chamber.

Analysis software was used to determine when individual paws are in contact with the belt of the treadmill to calculate several gait parameters. The gait parameters analyzed were stride time (the time between two initial paw contacts with the treadmill for the same paw), swing time (the part of stride time when the paw does not touch the treadmill) and stance time (the part of stride time when the paw does touch the treadmill). The analysis also included propelling time (the duration between maximum paw contact to the start of the swing phase) and braking time (the duration between the beginning of the swing phase and returning to maximum paw contact with the belt). The propel-to-brake ratio was then calculated. It is important to note that stride time = stance time + swing time and stance time = brake time + propel time. Measures of gait symmetry were also analyzed, including stride length and absolute paw angle (the angle that each paw makes in comparison to the long axis of the animal). Stance width (distance between forepaws and distance between hindpaws at peak stance) and paw area at peak stance were also assessed. For each mouse, the left and right paw values were averaged to give the fore and hind paw values as previously published (Pallier et al., 2009; Wright et al., 2015).

#### Gastrointestinal measures

##### Fecal output & water content

Fecal output was determined by single housing the animals for 1 hour and counting the number of excreted pellets. These pellets were collected, and the total weight was recorded before being dried at 95°C for > 3 hrs. The percentage difference between the initial total feces weight and the dry weight is taken as the fecal water content.

##### Gastrointestinal transit time

Gastrointestinal transit time was assessed using non-absorbable carmine red dye (Sigma-Aldrich), prepared as a 6% (w/v) dilution in 0.5% methylcellulose (Sigma-Aldrich), autoclaved and filtered before administration. Non-fasting mice were gavaged with 150 μL of the carmine red solution and housed individually. The time taken from gavage to the first appearance of the carmine red solution in the feces was recorded as the total gastrointestinal transit time.

##### FITC-dextran intestinal permeability

Intestinal epithelial barrier permeability was determined using 4 kDa fluorescein isothiocyanate (FITC) dextran (Sigma-Aldrich). Mice were fasted for 4hrs, then orally gavaged with 150 μL FITC-dextran, dissolved in PBS to a concentration of 100 mg/mL. Blood was collected via cardiac puncture 4hrs after administration, immediately transferred to an EDTA collection tube and centrifuged at 1,000 × g for 10 min. Plasma was then collected and fluorescence was quantified at an excitation wavelength of 485 nm and an emission wavelength of 528 nm (PHERAstar *FSX*, BMG Labtech). FITC-dextran serially diluted in PBS was used to calculate a standard curve.

##### Macroscopic measures

Mice were euthanized by cervical dislocation and their intestines were removed. The intestine was placed on a non-absorbent surface and the length of the cecum and colon were measured using a ruler. The cecum was then weighed. Macroscopic measurements were normalized to the body weight (g) of the mice unless otherwise stated.

##### SCFA and BCFA extraction and analysis

Tubes containing fecal samples of 10-30mg were randomized before the analysis. 400 μL of 50% acetonitrile and 4μL of 4-methylvaleric acid was added to each sample, then vortexed for 30 seconds. Samples were then incubated on a thermomixer at 10°C for 30 minutes at 950rpm before centrifuged at 14,000 rpm for 5 minutes at 4°C and the supernatant was extracted into a fresh tube for analysis. Pooled biological quality controls were run every five samples by creating a pooling extract (30μL) from individual study samples. Reagent blanks were analyzed to perform background correction and the reagent blank was then subtracted from the raw data. Internal standard normalization was conducted to account for the individual sample and preparation variations, and weight normalization was also performed to account for sample weight variations.

##### Fecal sample collection and DNA extraction for 16S rRNA sequencing

Fecal samples were collected at 12 weeks of age. Mice were housed individually for up to 10 minutes and fresh pellets were collected and immediately frozen in dry ice and stored at −80°C until further processing. The fecal genomic DNA was extracted using the PowerSoil HTP kit (Qiagen). The extracted genomic DNA was then amplified using prokaryotic 515F and 806R primers, targeting the V4 hypervariable region of the 16S rRNA gene. Amplicon 16S rRNA gene sequences were created using paired-end 150 bp sequencing on the Illumina MiSeq platform.

##### Bioinformatics and data pre-processing

Illumina MiSeq sequence raw FASTQ data were processed using Qiita for quality control, demultiplexing sequences and trimming. The final number of samples for the subsequent analysis was 95 (7 mice in the female-HD-EE group and 8 mice in all the other groups, average of 3 mice per cage), with a total of 5,139 operational taxonomic units (OTUs). Taxonomy classification of the OTUs was performed using the dada2 R package (Callahan et al., 2016) using the reference database "silva_nr99_v138.1_wSpecies_train_set.fa.gz". For alpha diversity analysis, reads were rarefied to 15,000. For beta diversity analysis (i.e., Bray-Curtis and unweighted UniFrac distances), we used non-rarefied counts. When calculating the unweighted UniFrac distances, we constructed an unrooted phylogenetic tree with a random root (dada2 R package). For multivariate analysis with sPLS-DA, mixOmics R package (Lê Cao et al., 2016; Rohart et al., 2017), counts were not rarefied, and males and females data sets were pre-filtered separately to remove OTUs with low counts across all samples (OTUs with sum counts of less than 0.01% of the sum of all counts were removed), resulting in 290 OTUs for females and 304 OTUs for males. The filtered counts were then transformed using CLR to account for the compositional data while calculating the relative abundance (Lê Cao et al., 2016). The CLR data were input into methods such as sPLS-DA, PCA and LMM described below.

### Quantification and statistical analysis

#### Statistical analysis of clinical variables

Single time-point observations, such as Macroscopic Measures, were modelled using Linear Mixed Models (LMM).

A LMM is an extension of a linear model to incorporate both fixed-effect terms and random effect terms. It is particularly useful when there is some dependence in the data. In this study, mice are housed within cages (random effect), while we are investigating the effect of sex (male, female), genotype (WT, HD) and housing (SH, EE, EX) (fixed effects). For repeated measures, time was added as a covariate. All two-way interactions between fixed effects were included. The resulting beta values represent the coefficient estimates of the fixed effects and their interactions. The sign and value of each beta coefficient indicate the effect of a particular covariate (e.g., sex) on the response while keeping other covariates constant (e.g., genotyping, housing, week). The effect of a particular variable reported in the LMM cannot be fully visualised in a graph while keeping other covariates and their interactions constant and controlling for random effects. Thus, to graph the main effect for each covariate, we corrected the data by subtracting the estimated interaction effects estimated from the LMM. For the variable clasping score measured on an ordinal scale, we fitted a cumulative linear mixed model with Laplace approximation. For models with at least one significant effect, we performed a post-hoc contrast analysis on estimated marginal means to identify significant pairs using the emmeans R package (Lenth et al., 2019). The emmeans method performs pairwise comparisons on the levels of significant main effects while controlling for other factors. For example, if genotype is found significant in the LMM, a pairwise comparisons between two genotypes separately for each sex and housing type is conducted. All p-values were then adjusted for multiple comparisons using Tukey adjustment. The significance level was set to 0.05.

#### Statistical analysis of 16S rRNA data

Stacked bar plots were then generated at the family level for each sample by normalizing counts to 1. Alpha diversity metrics, including species richness (Observed), Shannon and Inverse Simpson metrics were calculated on the rarefied OTU counts, using the Phyloseq R package (McMurdie and Holmes, 2013). While the observed species richness only takes the number of OTUs into account (i.e., richness), Shannon and Inverse Simpson diversity index take both richness and the relative abundance of OTUs (i.e., evenness). However, compared to the Shannon index, the Inverse Simpson is less sensitive to rare species as they are assigned a lower weight. One-way ANOVA was used to compare the species richness and alpha diversity measurements between the genotype, housing condition and sex.

Beta diversity was calculated with Bray-Curtis distance and unweighted UniFrac distance and visualised with PCoA. Bray-Curtis distance accounts for the abundance between OTUs, where unweighted UniFrac distance is based on the phylogenetic relationship between the OTUs. To assess whether there was any difference between housing conditions, sex and genotypes, Adonis (Permutation multivariate ANOVA - PERMANOVA) from the vegan R package was performed with 999 permutations (Anderson, 2001; Dixon, 2003).

To identify microbial drivers discriminating particular housing conditions in a specific sex and genotype group, we applied sPLS-DA (Lê Cao et al., 2011, 2016; Rohart et al., 2017) with two components. sPLS-DA is a supervised classification framework for discriminant analysis based on partial least squares regression with a Lasso penalization for variable selection. sPLS-DA assumes that only a subset of original predictors is driving the outcome variable. This assumption stands when the number of predictors is far more than the number of samples: a common attribute in microbiome data (Chung and Keles, 2010; Ruiz-Perez et al., 2020). sPLS-DA is a multivariate non-parametric method that does not require any distributional assumption about the data. The optimal number of OTUs selected on each component was determined using 5-fold cross-validation repeated 10 times. PCA sample plots were also examined to assess potential cage effects. No strong cage effects were observed (Figure S5). Within each sex, and for each OTU, we fitted an LMM with genotype and housing as fixed effects and cage as random effects to identify which fixed effects and their interactions may explain OTU abundance. p values were adjusted for multiple testing using the Benjamin and Hochberg (BH) procedure (Benjamini and Hochberg, 1995) in the stats R package (R Core Team, 2021).

All statistical and pre-processing analyses were performed using R software (version 4.1.0), with the use of R packages dada2 V1.20.0, Phyloseq V1.36.0, vegan V2.5, mixOmics V6.16.1 and stats V4.1.0. Graphs were created using both R and Graph® Pad Prism 9 software.

## Data Availability

The datasets and metadata related to this study have been deposited in the NCBI Sequence Read Archive under BioProject number PRJNA770470. Furthermore, the reproducible R code and report for the statistical analysis has been uploaded to a Github repository - https://github.com/SarithaKodikara/Gene_environment_gut_interactions_in_Huntington-s_disease.

## Supplemental information

Document S1. Figures S1–S5

Table S1. Post-hoc results for sex, genotype, and housing in variable weight across different weeks, related to Figure 1A

Table S2. Post-hoc results for sex, genotype, and housing in variable water intake across different weeks, related to Figure 1B

Table S3. Post-hoc results for genotype and housing given sex and week in rotarod variable, related to Figure 1C

Table S4. Post-hoc results for genotype given sex and housing types in variable forepaw propel brake ratio, related to Figure 1D

Table S5. Post-hoc results for genotype given sex and housing types in variable forepaw swing time, related to Figures 1D and S1A

Table S6. Post-hoc results for genotype given sex and housing types in variable hindpaw swing time, related to Figures 1D and S1A

Table S7. Post-hoc results for genotype given sex and housing types in variable forepaw stance width, related to Figures 1D and S1C

Table S8. Post-hoc results for genotype given sex and housing types in variable forepaw stride length, related to Figures 1D and S1D

Table S9. Post-hoc results for genotype given sex and housing types in variable hindpaw stride length, related to Figures 1D and S1D

Table S10. Post-hoc results for sex and genotype given housing type and week in variable clasping score, related to Figure 1E

Table S11. Post-hoc results for genotype and sex given housing type in variable brain weight, related to Figure 1F

Table S12. Post-hoc results for sex given housing type in variable Butyrate, related to Figure 2C

Table S13. Post-hoc results for sex given housing type in variable Valerate, related to Figure 2D

Table S14. Loading values for OTUs selected in sPLS-DA for females along with their adjusted p-values from LMM, related to Figure 7

Table S15. Loading values for OTUs selected in sPLS-DA for males along with their adjusted p-values from LMM, related to Figure 6

Table S16. Taxonomy details corresponding to OTUs in Tables S14 and S15, related to Figures 6 and 7 Note that all OTUs belonged to the Bacteria kingdom.
