# Supplementary material for: Gene-environment-gut interactions in Huntington's disease mice are associated with environmental modulation of the gut microbiome
Source: iScience. 2021 Dec 24;25(1):103687. doi: 10.1016/j.isci.2021.103687 (PMC8760441; doi:10.1016/j.isci.2021.103687)
Supplement: Document S1. Figures S1–S5 [file mmc1.pdf]

iScience, Volume *25*

Supplemental information

Gene-environment-gut interactions in Huntington's disease mice are associated with environmental modulation of the gut microbiome

Carolina Gubert, Chloe Jane Love, Saritha Kodikara, Jamie Jie Mei Liew, Thibault Renoir, Kim-Anh Lê Cao, and Anthony John Hannan

SUPPLEMENTAL INFORMATION

SUPPLEMENTAL FIGURES AND TABLES

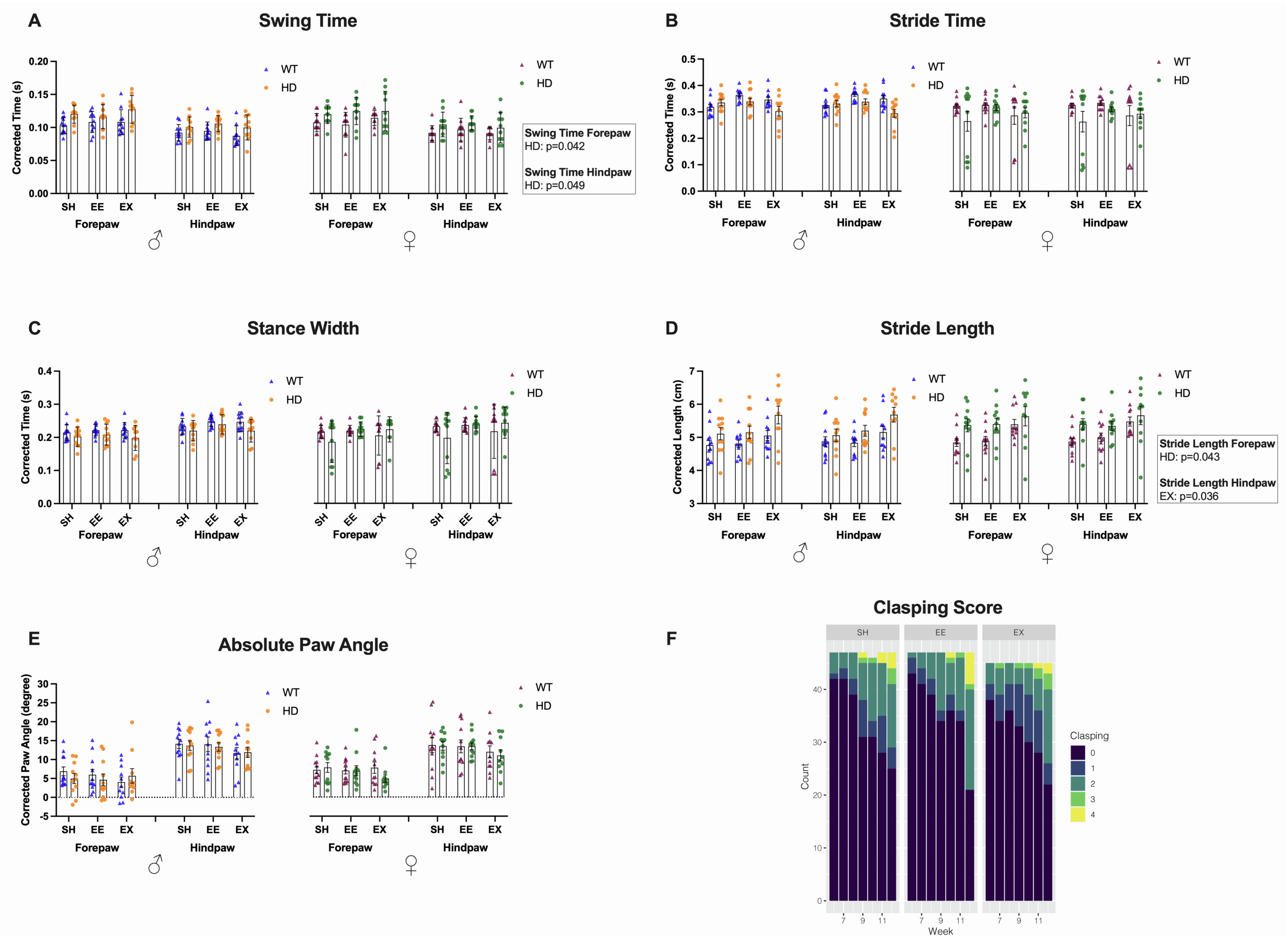

Figure S1. Digigait swing and stride times, stance width, stride length and absolute paw angle at 12 weeks of age as well as clasping scores by housing. Related to Figure 1. (A) HD mice showed an increased forepaw and hindpaw swing time than WT mice, LMM, n=10-12 mice. **(B)** No effects of housing, genotype or sex were seen in stride time, LMM, n=10-12 mice. **(C)** HD mice showed a decrease in forepaw stance width than WT mice and males have a greater stance width than females, LMM, n=10-12 mice. **(D)** HD mice showed an increase in forepaw stride length than WT mice. Mice housed in EX had an increased hindpaw stride length than mice in SH, LMM, n=10-12 mice. **(E)** No effect of housing, genotype or sex were seen in stride length or absolute paw angle, LMM, n=10-12 mice. **(F)** No effect of housing conditions was seen in clasping score, cumulative linear mixed model, n=11-12 mice. Data were corrected for interaction effects. Mean ± SEM are represented. WT, wild-type; HD, Huntington's disease; SH, standard housing; EE, environmental enrichment; EX, exercise; LMM, linear mixed model.

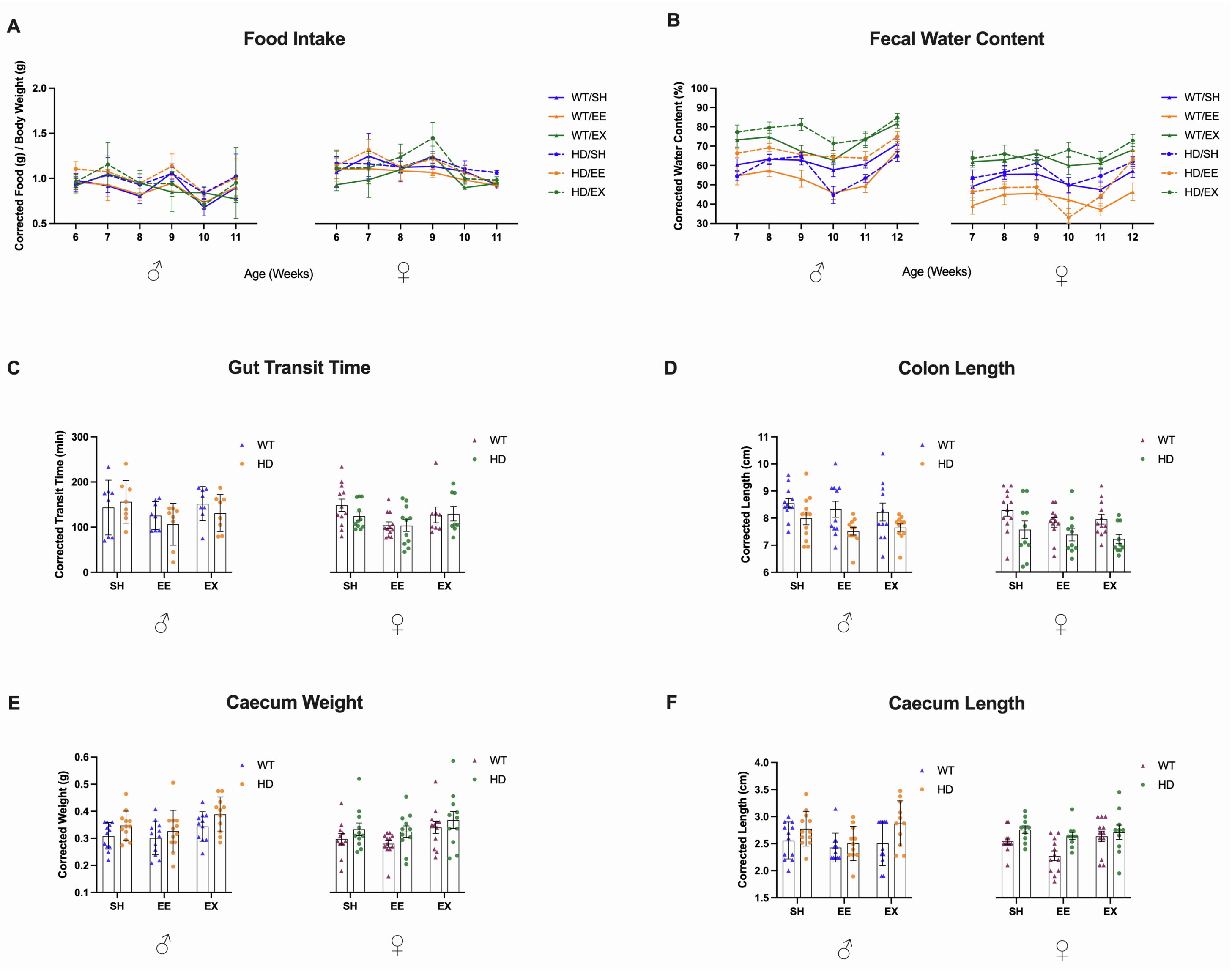

Figure S2. Effects of housing, genotype and sex on food intake, fecal water content, gut transit time, colon length, caecum weight and caecum length. Related to Figure 2. (A) No differences were observed in food intake, LMM, n=2-3 cages per group. **(B)** No effect of housing, genotype or sex on fecal water content was observed, LMM, n=8-12 mice. **(C)** No effect of housing, genotype or sex on gut transit time was observed at 12 weeks of age, LMM, n=8-13 mice. **(D-F)** No effects of housing, genotype or sex on gut macroscopy measures of colon length, caecum weight and caecum length at 12 weeks of age, LMM, n=10-12 mice. Data were corrected for interaction effects. Mean ± SEM are represented. LMM, linear mixed model.

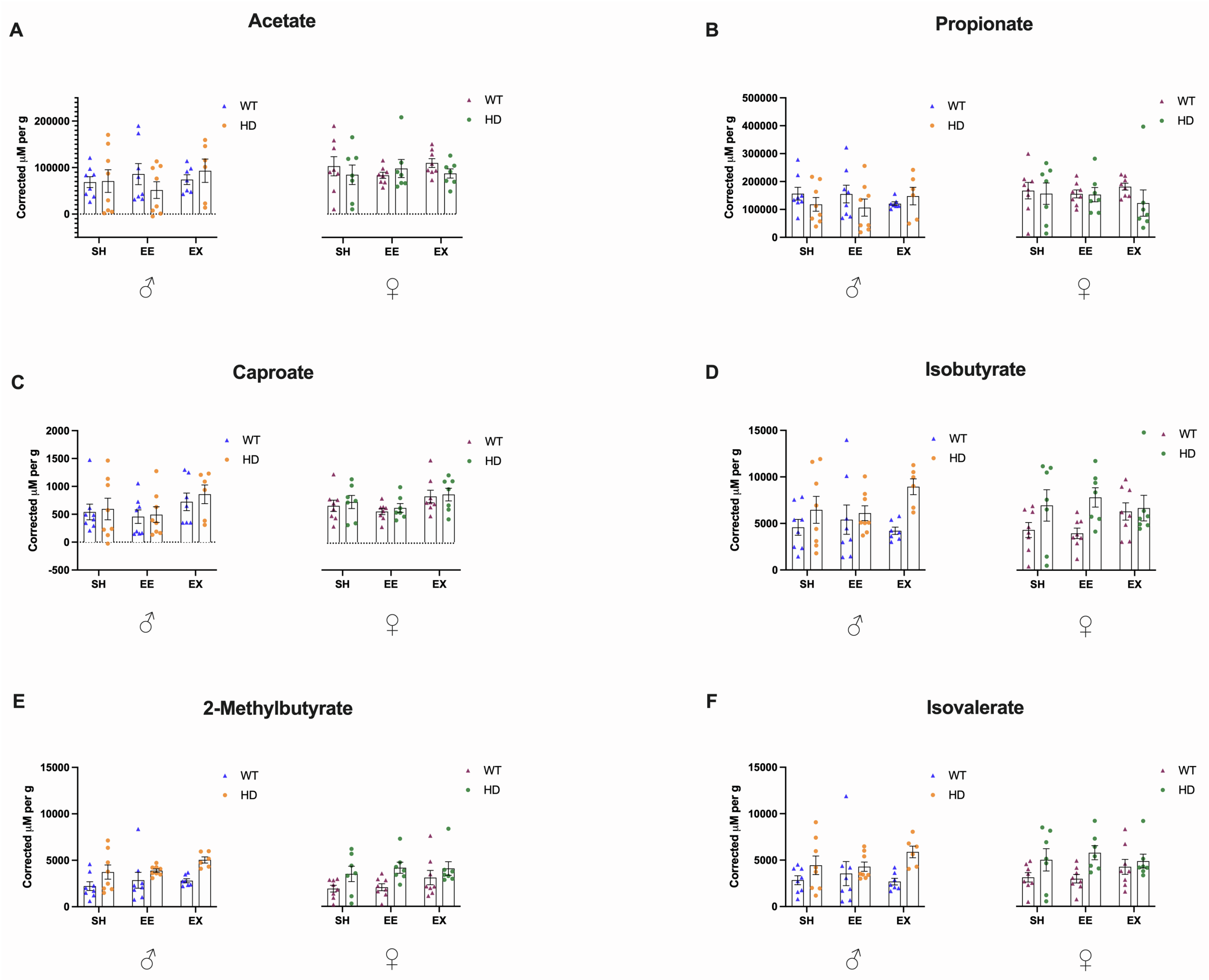

Figure S3. Effects of housing, genotype and sex on SCFA and BCFA concentrations. Related to Figure 2. (A-C) At 12 weeks of age, no differences were seen in SCFA concentrations of acetate, propionate or caproate. **(D-F)** At 12 weeks of age no differences in BCFA concentrations of isobutyrate, 2-methylbutyrate or isovalerate. Data were corrected for interaction effects. Mean ± SEM are represented, LMM, n=7-8 mice. SCFAs, short-chain fatty acids; BCFA, branched-chain fatty acids; WT, wild-type; HD, Huntington's disease; SH, standard housing; EE, environmental enrichment; EX, exercise; LMM, linear mixed model.

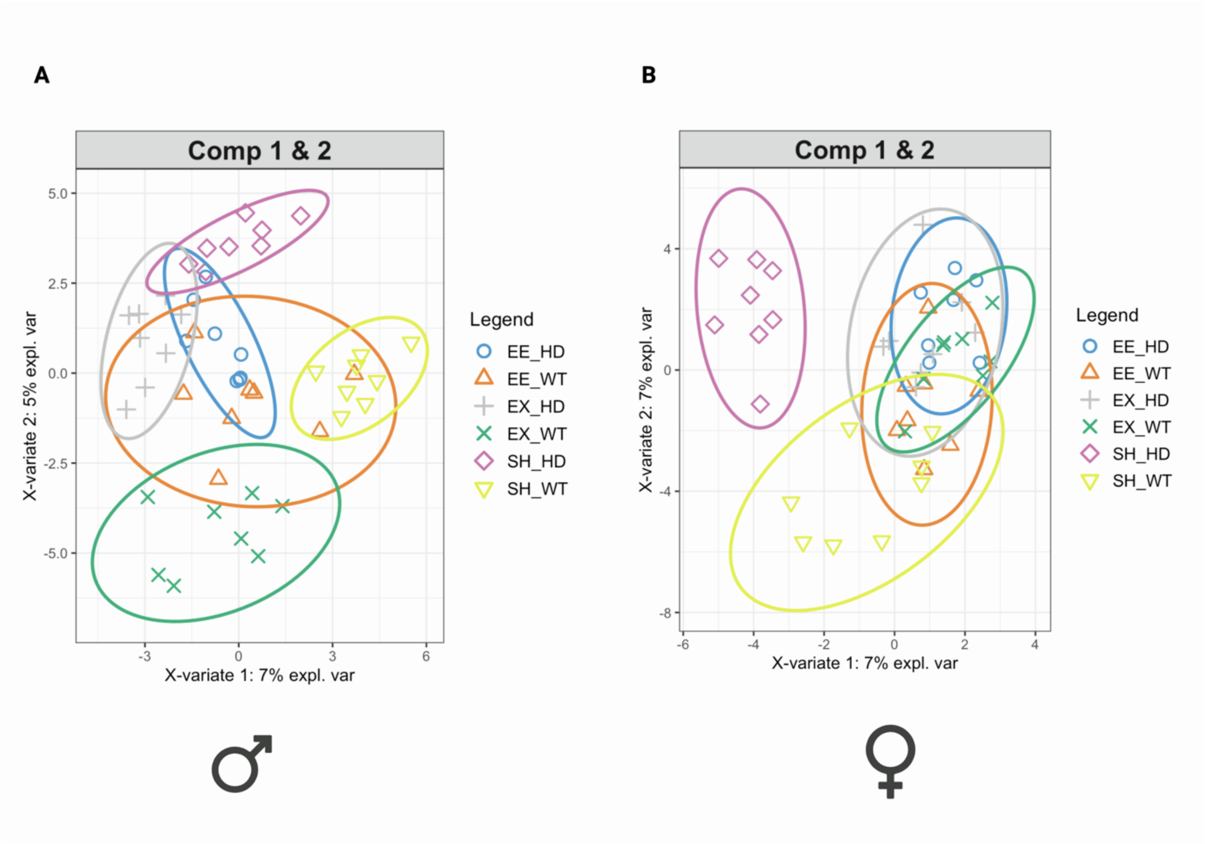

Figure S4. Identification of a bacterial signature in males and females discriminating housing conditions and genotype with sPLS-DA. Related to Figure 6. (A) Sample plots with 0.95 confidence ellipse plots show discrimination between genotype and housing conditions, showed no clear separation in both **(A)** males and **(B)** females. Overall classification error rates resulting from five-fold cross-validation repeated 10 times were 0.47 for males and 0.50 for females (all housing and genotypes). n= 7-8 samples per group. WT, wild-type; HD, Huntington's disease; SH, standard housing; EE, environmental enrichment; EX, exercise; OTU, Operational taxonomic units; sPLS-DA, sparse partial least squares regression-discriminant analysis.

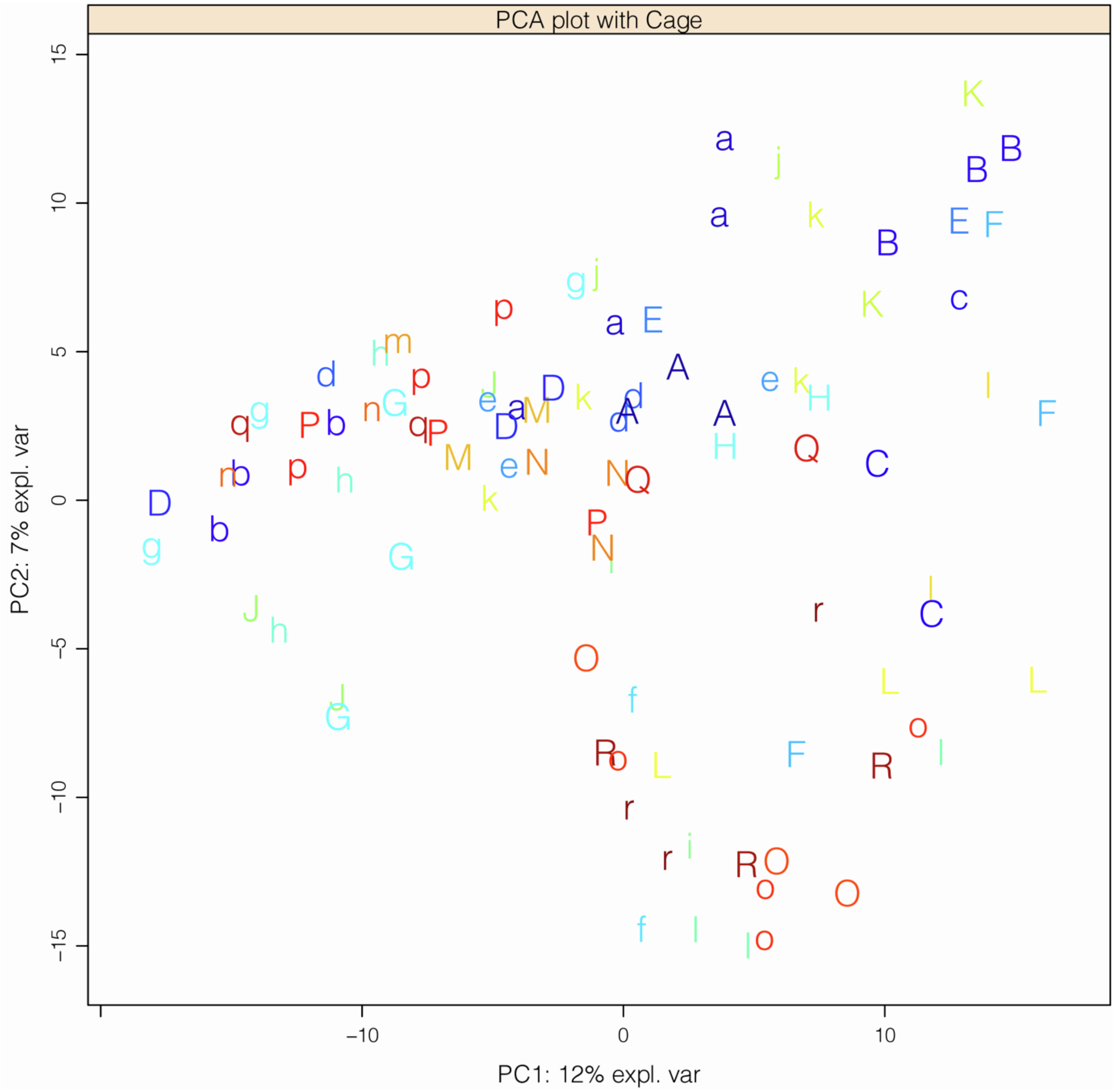

Figure S5. PCA sample plots assessing potential cage effects. **Related to STAR methods.** No strong cage effect was observed but only mild trends of cages clustering together (i.e., cages A, B). Colours and letters represent different cages, with upper case letters corresponding to cages with males and lower-case letters corresponding to cages with females. n = 3 cages per group. PCA, principal component analysis.
